# Special Issue “Microglia Heterogeneity and Its Relevance for Translational Research”

**DOI:** 10.3390/ijms222212350

**Published:** 2021-11-16

**Authors:** Alessandro Michelucci, Veronique E. Miron, Josef Priller

**Affiliations:** 1 Neuro-Immunology Group, Department of Oncology, Luxembourg Institute of Health, L-1526 Luxembourg, Luxembourg; 2 MRC Centre for Reproductive Health, The Queen’s Medical Research Institute, The University of Edinburgh, Edinburgh EH16 4TJ, UK; v.miron@ed.ac.uk; 3 Centre for Discovery Brain Sciences, Chancellor’s Building, The University of Edinburgh, Edinburgh EH16 4SB, UK; josef.priller@charite.de; 4 United Kingdom Dementia Research Institute, The University of Edinburgh, Edinburgh EH16 4SB, UK; 5 Department of Neuropsychiatry and Laboratory of Molecular Psychiatry, Charité-Universitätsmedizin Berlin, 10117 Berlin, Germany

Microglia are necessary for the development and function of the central nervous system (CNS). Their ontogeny, together with the particular environment of the CNS, make microglia a rather distinct population of neural cells [1]. The exceptional properties of microglial cells throughout the lifespan, such as their role in synaptic stripping or the notable capacity to scan the brain parenchyma and rapidly react to its perturbations, have emerged during the past years. Recent technological advances in molecular biology, imaging, and single-cell analyses are providing fascinating insights into the dynamic changes and diversity of microglia in the healthy, aging and diseased CNS, revealing their potential as therapeutic targets [2,3,4].

In this context, this Special Issue aims to provide a glimpse of our current understanding of the diversity of microglia spanning from regional heterogeneity under homeostasis to emerging disease-associated microglia with distinct transcriptional profiles reflecting specific activation states under pathological settings, including tumorigenic and degenerative processes. Thus, we collected articles that covered, but were not limited to, the role of microglia in inflammatory processes associated with CNS diseases as well as strategies for patient stratification, diagnostics or treatment of CNS disorders based on dynamic molecular changes in microglia.

In this Special Issue, four original articles and three reviews cover several aspects of microglial biology and heterogeneity, ranging from their specific adaptations in Alzheimer’s and Parkinson’s disease patients and their emerging diversity uncovered by the application of single-cell technologies under healthy conditions and various neurological illnesses.

In particular, Sorrentino and collaborators carry out both a neuropathological and biochemical analysis of Alzheimer’s disease (AD) brain samples. Altered microglial morphologies, densities and distribution were noted in AD. Measuring the brain levels of 25 inflammatory mediators allowed stratification of AD patients into three distinct “neuroinflammatory clusters”, suggesting that specific microglia profiles might be associated with characteristics and severity of AD [5].

Reimers and collaborators characterized “apoptosis-associated speck-like protein containing a caspase recruitment domain” (ASC), which modulates the activation of the inflammasome, in a transgenic mouse model of AD. They showed significant amounts of ASC in microglia and astrocytes associated with amyloid-beta (Aβ) plaques in aged transgenic mice, along with foci of clustered extracellular ASC granules, which were not associated with Aβ plaques [6].

Grieco and colleagues investigated lipid signalling in AD, focusing on the role of fatty acid amide hydrolase (FAAH), an integral membrane enzyme that hydrolyzes the endocannabinoid anandamide and related amidated signalling lipids. They found that the FAAH inhibitor URB597 applied to a mouse model of AD-like pathology altered cytoskeleton reorganization, regulated phagocytosis and cell migration, and skewed microglia towards an anti-inflammatory phenotype [7].

Badanjak and colleagues highlight recent findings on microglia–neuronal crosstalk in Parkinson’s Disease (PD), focusing on human post-mortem immunohistochemistry and single-cell studies, their relation to animal and induced pluripotent stem cell (iPSC)-derived models, newly emerging technologies and the resulting potential of new anti-inflammatory therapies for PD patients [8]. They discuss how PD may be driven by a vicious cycle between dying neurons and microglial activation through oxidative stress, mitophagy and autophagy dysfunctions, α-synuclein accumulation and pro-inflammatory cytokine release.

Timis and co-workers review the most significant findings related to microglia roles in intracranial aneurysms and vascular malformations, representing critical causes of intracranial haemorrhage and subsequent morbidity and mortality [9].

Interestingly, in a more technically oriented study, Han and colleagues demonstrate that experimental microglial depletion using both conditional genetic *Cx3cr1*^CreER/+^*Rosa26*^DTA/+^ animal models and pharmacological colony-stimulating factor 1 receptor (CSF1R) inhibitors also affect circulating monocytes and peripheral tissue macrophages, thus advising that effects on peripheral immunity should be considered when conducting microglial depletion studies, especially if taking them into account for immunotherapies [10].

Lastly, Ochocka and Kaminska review recent advances in single-cell approaches that allowed the study of microglia at high resolution, revealing a spectrum of discrete states, both under homeostatic and pathological conditions. In particular, single-cell technologies, including single-cell RNA sequencing (scRNA-seq) and mass cytometry (Cytometry by Time-Of-Flight, CyTOF) recently uncovered the heterogeneity of microglia and immune infiltrates in brain pathologies, such as neurodegenerative disorders, stroke, depression, and brain tumours [11].

Taken together, the present Special Issue contributes to our current understanding of the cellular and molecular mechanisms underlying microglia diversity within the healthy CNS and under inflammatory, neurodegenerative and tumorigenic conditions.

The application of single-cell techniques to phenotype homeostatic and activated microglia in different neurological disorders, combined with the functional screening of inferred cellular diversity, is currently opening new avenues to understand cellular and functional states in a context-dependent manner. In this regard, updated single-cell sequencing methodologies, including INs-seq [12] or CITE-seq [13] allowing protein markers to be overlaid onto scRNA-seq profiles, enable the integration of transcriptional states and immune phenotypes. Furthermore, RNA sequencing techniques with spatial resolution, such as Slide-seq [14], seek to link transcriptomic information with spatial distribution. Approaches also exist to analyse a specific function, such as Tox-seq for reactive oxygen species production and ascribe it to specific immune cell clusters [15]. Bioinformatics tools, such as NicheNet [16] and CellPhoneDB [17], enable the identification of receptor–ligand pairs from scRNA-seq data, which provide the basis to dissect the crosstalk between microglia and their surrounding cellular niche [18]. With these and other available tools, it will be conceivable to functionally characterise distinct microglial states in situ and subsequently manipulate them to, for example, render specific microglia subsets neuroprotective or anti-tumorigenic. In line with this notion, a disease-associated microglia subset, which can be therapeutically modulated, has been recently identified in AD [19]. Lastly, for translational ends, to further investigate the relevance of the identified microglia states and diversity in human diseases, it will be critical to employ human-relevant models, such as iPSC-derived cells. These models will be ultimately instrumental for functional analyses and high-throughput drug screening aimed at modulating microglia state activities according to specific brain diseases.
